# Impact of Petroleum Exposure on Some Hematological Indices, Interleukin-6, and Inflammatory Markers of Workers at Petroleum Stations in Basra City

**DOI:** 10.1155/2020/7693891

**Published:** 2020-08-04

**Authors:** Azza Sajid Jabbar, Eman T. Ali

**Affiliations:** ^1^Department of Pharmacology and Toxicology, College of Pharmacy, University of Basrah, Basra, Iraq; ^2^Department of Clinical Laboratory Sciences, College of Pharmacy, University of Basrah, Basra, Iraq

## Abstract

**Background:**

Occupational and environmental exposure to several pollutant factors such as petroleum products containing benzene has toxic effects on different body systems. The hematopoietic system and immune system are among the affected systems. This study aims to investigate the effect of benzene exposure on some blood parameters of workers at several fuel stations in Basra city, as well as to reveal if the continuous exposure may induce an inflammatory response, which is reflected by changes in some hematological and inflammatory markers.

**Methods:**

The study included two groups of males. The first group consists of 72 exposed workers at petrol stations in different locations in the Basra city. The other group is the control group, which consists of 75 nonexposed subjects (students and faculty members of the college). Different hematological parameters (WBC, RBC, HGB, MCV, MCHC, and MCH) have been evaluated. Serum concentrations of IL-6 and hs-CRP were estimated in all workers and nonexposed using enzyme-linked immunosorbent assay (ELISA).

**Results:**

Data showed significant hematological changes in the exposed workers, and that anemia was a common disorder among them. Furthermore, there was a significant decline in WBC and different types of WBC including lymphocytes, monocytes, and neutrophils in the exposed workers. Erythrocyte sedimentation rate and serum levels of interleukin-6 and hs-CRP were significantly higher in exposed workers than in nonexposed. A significant correlation was identified among blood parameters, while a strong inverse correlation was identified between both MCHC and ESR. The most significant inverse correlation was found between RBC and IL-6 and MCH with hs-CRP. In addition, a significant negative correlation was found between monocytes and IL-6.

**Conclusion:**

The changes in all hematology and inflammatory parameters refer to damage in the hematopoietic system due to continuous exposure to vapors of petrol products, which also result in a significant increase in interleukin-6.

## 1. Introduction

Petroleum station is a source of a mixture of hydrocarbon compounds, aliphatics, aromatics, such as benzene and toluene, and hundreds of saturated and unsaturated hydrocarbons. Therefore, the gases and vapors emitted from petroleum stations may contribute to various forms of pollutions. The continuous exposure to the vapors of this mixture and working conditions at occupational places makes the workers at risk of various diseases that attack different body systems such as the respiratory system [1], cardiovascular system, immune system, and renal system [2]. Furthermore, the harmful effect due to the toxicity including immunotoxicity and neurotoxicity may make these workers more susceptible to inflammatory diseases and injuries. It was approved that toxicity of inhaled vapors including benzene is due to its biotransformation to reactive oxygen species. Benzene is metabolized in the liver to phenol, which in turn is exposed to hydroxylation to form hydroquinone and 1,4-benzenetriol by peroxidase of the bone marrow or by autoxidation [3]. Different mechanisms explain benzene-induced toxicity; however, the effect of benzene on health has been established as a carcinogenic contaminant by the International Agency for Research on Cancer [4]. Benzene and its metabolites have toxic effects on the hematopoietic system that lead to bone marrow suppression [5]. The increased susceptibility to injuries and infections because of leucopoiesis suppression is the major toxic effect of benzene among workers who were at a continuous exposure to benzene [6]. Moreover, benzene metabolites can activate the formation of cytokines and chemo-cytokines by peripheral blood mononuclear cells (PBMC) [7]. Moreover, infections and inflammatory processes may activate cytokines mediating immune response [8]. The imbalance of pro and anti-inflammatory molecules has been detected in serum of humans who are in continuous exposure to air pollutants [9]. Cytokines are mediators formed in response to different processes in the body such as growth, defense, and repair processes. They are a class of low molecular weight proteins, small molecules secreted by T helper cells (Th), and macrophages. Their release activates a cascade of reactions leading to systemic inflammation and consequent events [10, 11]. They regulate the immune and inflammatory responses [10]. Interleukin 6 (IL-6) is an important cytokine with multiple effects. It is a protein of a low molecular weight secreted mainly by cells of the immune system (monocytes and macrophages) to regulate various biological processes, such as proliferation, differentiation, and synthesis of other proteins [12]. On the other hand, IL-6 stimulates inflammatory processes, including B lymphocyte differentiation [12]. This study was constructed to investigate the effect of exposure to petrol vapor containing benzene on some blood parameters of workers at several petroleum stations in Basra city, as well as to show if this exposure may result in inflammatory process, through measuring of some inflammatory markers such as neutrophil and monocytes numbers, IL-6, and hs-CRP in male workers who are exposed to the vapors for more than 5 years.

## 2. Materials and Method

### 2.1. Study Design

The study was conducted in Basra City, South of Iraq, for the period between November 2018 and April 2019. The source of the involved subjects was divided into two groups: the first group consists of 72 exposed workers who worked in petrol stations in different locations of Basra city. All workers participating in this study have worked at petrol stations for not less than five years. The duration of exposure was at a rate of 12 hours per day for 4 days a week. The workers were with a range of 21–42 years of age, and the mean value is 32.71 ± 6.07. The second group consists of 75 nonexposed subjects (students and faculty members). They were within the range of 20–41 years of age, and the mean value is 31.46 ± 6.9. All participants were male because females in Iraq do not normally work in fuel stations.

### 2.2. Questionnaire

The required information of the participants were obtained by a questionnaire, which included demographic details (age and gender), personal lifestyle (smoking and alcohol), occupational history and duration of exposure (years of work and number of hours per day), and health status (hereditary diseases, drugs taken, and other diseases such as blood diseases, renal diseases, diabetes mellitus, and hypertension). Data were collected at the beginning of the day at 9 am, and less crowded days were chosen to interview the workers.

### 2.3. Sample Collection

After obtaining approval from the participants orally, five milliliters of blood was drawn from each worker using a vein puncture approach and then collected in two tubes. The rest of the blood was contained in a gel plain tube to prepare the serum for further study. To avoid any effects of ambient temperatures on the samples, the collected blood samples were always stored in cold conditions with a wet icebox until the time of measurement. Samples were measured on the same day. All laboratory works were performed in a private laboratory for analyzes.

### 2.4. Hematology Profile

Two milliliters of venous blood in a tube containing EDTA (disodium ethylenediaminetetraacetate) have been used to measure the hematological indices.

#### 2.4.1. Complete Blood Count (CBC) Test

Hematological parameters making up the complete blood count (CBC), i.e., RBC, HGB (g/dl), MCV (fl), MCHC (g/L), MCH (pg) and total white blood cell count and differential count (lymphocytes, monocytes, and neutrophils) were performed using the auto hematology analyzer (Ruby, Germany). According to the WHO criteria, workers with hemoglobin value < 13 g/dL in men were diagnosed anemic.

#### 2.4.2. Erythrocytes Sedimentation Rate (ESR) Test

The nonspecific body-screening test and erythrocytes sedimentation rate (ESR) were measured by the Westergren method, the unit being millimeter per hour (mm/h) [13].

### 2.5. ELISA Assay

#### 2.5.1. Estimation of IL-6 Cytokine

By using enzyme-linked immunosorbent assay (ELISA), serum levels of IL-6 were estimated for all exposed workers at petroleum stations and nonexposed subjects according to the manufacturer's instructions (PeproTech, USA).

#### 2.5.2. Evaluation of (hs-CRP) Protein Level

This is a blood test that detects the low levels of C-reactive protein (CRP). This protein is a specific indicator test, which measures low levels of inflammation in the serum of exposed workers and nonexposed using ELISA according to the manufacturer's instructions (Demeditec, Germany). The reagents from this company were ready to use, i.e., chromogen solution, conjugate, stop solution, and MTP-international stander 5-vials excluding washing solution and specimen diluents. A standard optical density (OD) curve was obtained for each calibrator provided with the kit for the corresponding concentration values in mg/l. The samples of more than 3.0 mg/l are considered high risk.

### 2.6. Statistical Analysis

Statistical analysis of the data was performed using Statistical Package for the Social Sciences (SPSS) software for Windows, version 24.0, IBM (SPSS Inc., IL, USA). The data are represented as an average value ± standard deviation (SD). The comparison of two sets of differences in the distributed numerical variables was estimated by the independent Students' *t*-test (groups 1 and 2). The Kruskal–Wallis test was used to get an analysis of ESR results. The Pearson correlation test was used to find out the correlations between different laboratory findings. The significance level was measured by two-tailed paired. *P* values at levels (*P* < 0.05) were significant.

## 3. Results

### 3.1. Demographics of the Study

Table 1 shows that 72 exposed workers who worked at petrol stations for five years and more. Their mean age was 32.71 ± 6.07. On the other hand, the number of nonexposed (control group) in 75 subjects, with a mean of age of 31.46 ± 6.9 is also given. They did not expose to the vapors of the petrol station.

### 3.2. Comparison between the Exposed Workers at Petroleum Stations and Nonexposed Subjects in Hematological Parameters

Data analysis to compare between the exposed workers at petroleum stations and nonexposed subjects in hematological parameters showed significant changes, as given in Table 2. These changes included significant reductions in all RBC, HGB (g/dl), MCV (fl), MCH (pg), and MCHC (g/L) are (3.04 ± 0.77 vs 4.34 ± 2.14), (9.3 ± 2.1 vs 11.8 ± 1.5), (75.02 ± 5.84 vs 86.91 ± 6.06), (27.51 ± 2.80 vs 28.86 ± 2.48), and (28.83 ± 2.90 vs 32.25 ± 2.49), respectively, when compared.

### 3.3. Total WBC and Differential Count Comparison between Exposed Workers and Nonexposed Subjects Groups

Both total WBC count and differential count tests of WBC showed significant differences in the mean values between the exposed workers and nonexposed subjects (control group). These differences are specifically clear in the mean value of lymphocytes and neutrophils as given in (Table 3). Moreover, WBC mean value has decreased significantly at level *P*=0.0001 (4198.4 ± 337.5 vs 6478.1 ± 381.4). Lymphocyte's mean value has decreased significantly at *P*=0.0001 (1.46 ± 0.80 vs 2.47 ± 1.19). The same finding was reported to the mean value of neutrophils at *P*=0.0001 (3.06 ± 1.02 vs 5.00 ± 1.388). However, data revealed a significant difference in the mean value of monocytes between the two groups (Table 3).

### 3.4. Comparison of the Inflammatory Markers IL-6, hs-CRP, and ESR between Exposed Workers and Control Groups

Comparison of the inflammatory markers between the two studied groups revealed that each of ESR, hs-CRP, and IL-6 have increased significantly as given in Table 4, ESR (11.80 ± 3.04 vs 8.83 ± 1.75), hs-CRP (5.87 ± 1.51 vs 1.93 ± 0.80), and IL-6 (12.21 ± 2.80 vs 5.54 ± 1.80) (*P* ≤ 0.001).

### 3.5. Correlation Assessment between RBC and Hematology Profile

Based on our assessment of whether the laboratory values are interrelated, we found a significant correlation between the following parameters: RBC was positively correlated to HGB (*r* = 0.380^*∗*^, *P*=0.03) and MCV(*r* = 0.489^*∗∗*^, *P*=0.005), also it was a positive correlation between WBC and MCHC (*r* = 0.412^*∗*^, *P*=0.01). As well as, there was a highly significant correlation between MCHC and HGB (*r* = 0.454^*∗∗*^, *P*=0.009) as shown in (Table 5).

### 3.6. Correlation Assessment between Hematological Parameters and Inflammatory Markers

Data analysis revealed that there was a strong inverse correlation between MCHC and ESR (*r* = −0.601^*∗∗*^, *P*=0.0002). The most significant correlation we want to concentrate on is between RBC and IL-6 (*r* = −0.380^*∗*^, *P*=0.03), also MCH level was inversely correlated to hs-CRP (*r* = −0.388^*∗*^, *P*=0.02). On the other hand, the study confirmed the existence of a strong positive correlation between each of IL-6 and hs-CRP, as shown in Table 6.

### 3.7. Correlation Assessment between Immune Cells, Hematological Parameters, and IL-6

The results confirmed the existence of relationships between the immune cells and each of WBC, MCV, and IL-6, as given in Table 7. A direct positive correlation was found between lymphocytes and both WBC and neutrophils (*r* = 0.401^*∗*^, *P*=0.02 and *r* = 0.421^*∗*^, *P*=0.02), respectively, also a positive correlation was found between monocytes and neutrophils (*r* = 0.345^*∗*^, *P*=0.04), as well as between neutrophils and WBC. Furthermore, there is an inverse relationship between monocytes and inflammatory cytokine IL-6. Finally, the study found a strong positive correlation between both neutrophils and MCV as given in Table 7.

## 4. Discussion

This study was conducted to investigate the effect of petrol vapor containing benzene on inflammatory and immune markers of human, besides its effect on hematological parameters such as CBC measuring, which is the main assessment of benzene toxicity in human [14] in areas characterized by high temperature (south of Iraq). A previous study informed that environmental factors such as temperature may play a role in concentrating the pollutants that result in petroleum products' vapors [15]. Few studies have examined the early effect of low benzene exposure on both hematological and immunological markers in the human population. Moreover, these studies have concluded controversial findings due to various factors that may limit similar outcomes. These factors are represented by the differences in sample size, duration of exposure to petrol vapors, and applying different safety instructions by the workers due to different work laws and systems that regulate the work at petrol stations all over the countries. The harmful effects of petroleum products inhalation, including benzene and gasoline on human health particularly blood components have been established by several previous studies [15–17] in people who are chronically exposed to the vapor of petroleum, and these studies have found inconsistent results on blood components. Whereas some studies have concluded that benzene and gasoline exposure led to a reduction in blood components [11, 14, 15, 18], and other studies did not find significant changes [19].

Despite the small sample size applied in this study, we found significant differences between the two studied groups in hematological parameters, immunological, and inflammatory markers. Moreover, data revealed correlations between hematological indices and both inflammatory and immunological markers.

The induced inflammatory process due to petrol vapor exposure could reduce the hematological parameters significantly and stimulate some immune processes. It has been established that chronic exposure to benzene may damage the precursors, stem cells, and stromal cells resulting in bone marrow suppression due to the presence of toxic compounds that turn to oxidative compounds [5, 12]. In the current study, we found negative changes in different blood components, whether in RBC or WBC. The most common hematological disorders found in the study were anemia and leukopenia due to benzene exposure at petroleum stations. There was a significant decline in HGB, RBC, MCV, and MCHC of workers who were in continuous exposure to vapor of petroleum stations for 5 years and more. These findings were compatible with the results of other studies [15, 16, 18, 20], which concluded that the toxic effect of petroleum products could result in many hematological disorders. This may be explained by the fact that hydrocarbons such as benzene, a metal such as lead, and other volatile vapor may result in a pathogenic effect on the bone marrow and lymph nodes and then on blood parameters, and the toxic effect of these materials can inhibit and destroy the hematopoietic components in the red bone marrow [21].

The present study showed that exposure to low levels of benzene and gasoline (the main constituent of petrol vapors) resulted in significant changes in the studied parameters represented by a significant decline in WBC, which have protective functions against infectious diseases and foreign bodies [22]. This finding suggests that aromatic hydrocarbons may contribute to a significant decrease in WBC and different types of WBC [23]. This result is consistent with other previous studies [20, 24, 25]. The suppression in bone marrow function and the failure in the migration of phagocytic cells due to the continuous exposure to petroleum products could reduce the number of neutrophils and monocytes in a way that could affect the immune processes of the workers and may make them vulnerable and more susceptible to different infections [25]. It may also be attributed to changes in numbers of lymphocytes, peripheral blood mononuclear cells (PBMCs), and macrophages, impaired responses to mitogens, and depression of neutrophil functions [26]. This was confirmed by the results of our study through the existence of the strong positive relationship between lymphocytes and neutrophils. Furthermore, the result of the current study found significant differences in the mean value of lymphocytes between exposed workers at petroleum stations and the subjects of the other group. This result is consistent with many different studies that have found that exposure to benzene results in a reduction in both circulating B and T lymphocytes in vivo and reduction in mitogen-stimulated lymphocytes proliferation [27, 28].

We found that the level of IL-6 as a proinflammatory marker was significantly increased in the workers group compared to the nonexposed group. This is due to different pathological pathways which may be induced as a result of continuous exposure to the pollutants in the air [9]. Despite that the effect of benzene exposure on human health has been proven, the mechanism of benzene toxicity is still not completely understood [24]. However, the toxicity may act through different pathways [7]. These pathological pathways result in releasing different mediators and inflammatory molecules including cytokines [9, 12]. Their release by T-cells and macrophages may induce a series of reactions that result in systemic inflammation [29] and play a significant role in immune response [8].

This result is also supported by another study by Bruce Gillis et al. [30], which illustrated that benzene has a toxic effect on the hematopoietic system and may stimulate the production of proinflammatory IL-6 and suppression of anti-inflammatory cytokines such as IL-10. The ability of benzene to result in an imbalance in the immune system was concluded by other studies [31], and that benzene may damage the lymphokines producing system, which regulates both hematopoiesis and immunity [32].

The concentration of cytokines was observed to be changed in circulation; therefore, they have been considered as markers to evaluate the harmful effect of continuous exposure to petroleum products as major pollutants in the air [33]. The proposed mechanism due to the entry of toxic materials of the petroleum products includes various inflammatory processes and development of ROS [12] and one of the cytokines, which play a role in the pathological process is the IL-6 [34]. A previous study has concluded that specific cytokines producing cells were highly sensitive to the toxic effect of benzene; therefore, the immune system would become imbalanced, and that benzene can damage the system responsible for producing lymphokines and inhibit the immune function and hematopoiesis [33]. Another mechanism that has been assumed in our current study was that benzene can influence both cellular and humoral immune responses by altering Th cell function and increased sensitivity to autoimmune development, hypersensitivity, cancer, and infectious diseases [35]. In particular, benzene can improve the growth of helper T-cells 2 (Th2) that affect the proliferation of Th1 cells. Th2-mediated responses are responsible for humoral immunity that produces IL-6, which stimulates the differentiation and interaction of strong antibody responses but inhibits many phagocytic cell functions [36]. However, additional and further studies are required to confirm this hypothesis.

The current study has shown a significant elevation in hs-CRP in petroleum workers compared to unexposed suspects, which is in agreement with previous studies [37]. CRP is a classical acute phase protein that binds to ligands exposed to damaged tissues [38]. A shred of evidence proved that increased CRP production is characteristic of inflammatory, infective, and tissue damage [38]. Consequently, first, the current elevation in CRP may be due to the persistent inflammatory status resulting from exposure to benzene, and second, due to the high concentration of interleukin-6 that provokes the liver to form acute phase proteins, including the reactive protein (CRP) [12]. Our results confirmed this explanation by the presence of a significant positive correlation between IL-6 and hs-CRP.

We found a significant increase in the ESR value of the exposed workers compared to that of nonexposed subjects. This finding is an inevitable result of the increased inflammatory processes in exposed workers. This change is consistent with the finding of a previous study [2]. Raised ESR is said to be an indicator of general health defects of nonspecific nature. This is confirmed by our results that we found all exposed workers are with hematological disorders such as anemia. This result could be proven by the significant correlation between ESR and the hematological indices. However, the constantly elevated ESR among the exposed workers may suggest an occurrence of clinical disorders due to occupational exposure to a variety of toxic pollutants including petrol products vapors [39].

## 5. Conclusion

The main outcome that we may conclude from this study is the increased inflammatory processes among the petrol station workers who work for not less than five years. These inflammatory processes are represented by an increase in IL-6, ESR, and hs-CRP. Moreover, this occupational exposure could induce negative hematological changes and disorders such as anemia. However, further studies are required for a complete understanding of the mechanism of benzene exposure in causing immune toxicity and inflammatory response.

## Figures and Tables

**Table 1:** Demographics of the study.

Demographics	Exposed workers	Nonexposed workers
Age^*∗*^	32.71 ± 6.07 (42–21)	31.46 ± 6.9 (20–41)
Number	72	75
Duration of exposure	≥5	None
No. of hours per day	12 per day	None
Smoking	None	None
Hereditary diseases	None	None

^*∗*^ *P* is not significant at level <0.05. No.: number.

**Table 2:** Comparison between exposed workers at petroleum stations and nonexposed subjects in hematological parameters.

Groups	Hematological parameter				
	RBC (10^6^/*µ*L)	HGB (g/dl)	MCV (fl)	MCH (pg)	MCHC (g/L)
Workers	3.04 ± 0.77	9.3 ± 2.1	75.02 ± 5.84	27.51 ± 2.80	28.83 ± 2.90
Nonexposed subjects	4.34 ± 2.14	11.8 ± 1.5	86.91 ± 6.06	28.86 ± 2.48	32.25 ± 2.49
*P*≤	0.0001	0.0001	0.0001	0.04	0.0001

*P* is significant statistics at level < 0.05. Data are reported as mean ± standard deviation. RBC: red blood cells; HGB: hemoglobin; HCT: haematocrit; MCV: mean corpuscular volume; MCH: mean corpuscular hemoglobin; and MCHC: mean corpuscular hemoglobin concentration.

**Table 3:** Total WBC and differential count comparison between exposed workers and nonexposed subjects groups.

Groups	Differential count			
	WBC	Lymphocytes	Monocytes	Neutrophils
Exposed workers	4198.4 ± 337.5	1.46 ± 0.80	0.563 ± 0.244	3.06 ± 1.02
Nonexposed subjects	6478.1 ± 381.4	2.47 ± 1.19	0.733 ± 0.340	5.00 ± 1.388
*P*≤	0.0001	0.0001	0.02	0.0001

*P* is significant at level ≤0.05. WBC: white blood cells.

**Table 4:** Comparison of the inflammatory markers IL-6, hs-CRP, and ESR between exposed workers and nonexposed subjects groups.

Groups	Inflammatory makers		
	IL-6 (pg/mL)	hs-CRP (mg/L)	ESR (mm/h)
Exposed workers	12.21 ± 2.80	5.87 ± 1.51	11.80 ± 3.04
Nonexposed subjects	5.54 ± 1.80	1.93 ± 0.80	8.83 ± 1.75
*P*≤	0.0001	0.0001	0.0001

ESR: erythrocyte sedimentation rate; hs-CRP: high-sensitivity C-reactive protein, and IL-6: Interleukin-6.

**Table 5:** Correlation assessment between RBC and each of HGB, MCV, MCH, and MCHC among the workers group.

Correlation	Hematological parameters			
	RBC and HGB	RBC and MCV	MCHC and WBC	MCHC and HGB
*r*	0.543^*∗∗*^	0.489^*∗∗*^	0.412^*∗*^	0.454^*∗∗*^
*P*	0.001	0.005	0.019	0.009

^*∗∗*^Correlation is significant at the 0.01 level (2-tailed). ^*∗*^Correlation is significant at the 0.05 level (2-tailed).

**Table 6:** Correlation assessment between hematological parameters and inflammatory markers.

Correlation	RBC and IL-6	MCH and hs-CRP	MCHC and ESR	IL-6 and hs-CRP
*r*	−0.380^*∗*^	−0.388^*∗*^	−0.601^*∗∗*^	0.978^*∗∗*^
*P*	0.03	0.02	0.0002	0.0001

^*∗∗*^Correlation is significant at the 0.01 level (2-tailed). ^*∗*^Correlation is significant at the 0.05 level (2-tailed).

**Table 7:** Correlation assessment between immune cells, hematological parameters, and IL-6.

Parameters	*R*	*P*
Lymphocytes and WBC	0.401^*∗*^	0.023
Lymphocytes and neutrophils	0.421^*∗*^	0.02
Monocytes and IL-6	−0.426^*∗*^	0.01
Monocytes and neutrophils	0.345^*∗*^	0.04
Neutrophils and MCV	0.467^*∗∗*^	0.007
Neutrophils and WBC	0.493^*∗∗*^	0.004

^*∗∗*^Correlation is significant at the 0.01 level (2-tailed). ^*∗*^Correlation is significant at the 0.05 level (2-tailed).

## Data Availability

The data used to support the findings of this study are included within the article.
